# The prevalence of hip osteoarthritis: a systematic review and meta-analysis

**DOI:** 10.1186/s13075-023-03033-7

**Published:** 2023-03-29

**Authors:** Zijuan Fan, Lei Yan, Haifeng Liu, Xiaoke Li, Kenan Fan, Qiang Liu, Jiao Jiao Li, Bin Wang

**Affiliations:** 1 Department of Orthopaedic Surgery, The First Affiliated Hospital, Zhejiang University School of Medicine, Qingchun Road No. 79, Hangzhou, China; 2 Department of Health Statistics, School of Public Health, Shanxi Medical University, Taiyuan, China; 3 Department of Orthopaedic Surgery, Shanxi Medical University Second Affiliated Hospital, Taiyuan, China; 4 Arthritis Clinic and Research Center, Peking University People’s Hospital, Beijing, China; 5 School of Biomedical Engineering, Faculty of Engineering and IT, University of Technology Sydney, Sydney, Australia

**Keywords:** Prevalence, Radiographic, Hip osteoarthritis, Systematic review, Meta-analysis

## Abstract

**Objective:**

To estimate the global prevalence of hip osteoarthritis (HOA) through a systematic review and meta-analysis, and to determine by regression analysis the respective relationships between age and sex, and sex and prevalence.

**Methods:**

EMBASE, PubMed, Web of science, CINAHL, and SCOPUS were searched from inception until August 2022. Two authors independently extracted data and assessed the quality of the retrieved literature. Random-effects meta-analysis was performed to derive the pooled prevalence. Variations in the prevalence estimate in different subgroups, including diagnostic methods, region, and patient sex, were examined by subgroup meta-analysis. Meta-regression was used to construct the age-specific prevalence of HOA.

**Results:**

A total of 31 studies were included in our analysis, involving 326,463 participants. Quality evaluation showed that all studies included in the analysis had a Quality Score of at least 4. The most frequently used method for diagnosing HOA was the Kellgren–Lawrence (K-L) grade classification, accounting for 19/31 (61.3%) studies. The pooled prevalence of HOA diagnosed based on the K-L grade ≥ 2 criterion was 8.55% (95% CI 4.85–13.18) worldwide. The prevalence of HOA was lowest in Africa at 1.20% (95% CI: 0.40–2.38), followed by Asia at 4.26% (95% CI 0.02–14.93) and North America at 7.95% (95% CI 1.98–17.36), and highest in Europe at 12.59% (95% CI 7.17–19.25). There was no statistically significant difference in HOA prevalence between men (9.42%, 95% CI:4.81–15.34) and women at (7.94%, 95% CI: 3.57–13.81). The regression model showed a correlation between age and the prevalence of HOA.

**Conclusion:**

HOA has high prevalence worldwide and increases with age. The prevalence varies significantly by region but not by patient sex. High-quality epidemiological studies are warranted to more accurately estimate the prevalence of HOA.

**Supplementary Information:**

The online version contains supplementary material available at 10.1186/s13075-023-03033-7.

## Introduction

Osteoarthritis (OA) is a leading cause of disability and morbidity globally [1–3]. According to the Prevalence Trends of Site-Specific Osteoarthritis From the Global Burden of Disease Study 2019, prevalent cases of OA increased by 113.25% over a 10-year period, from 247.51 million in 1990 to 527.81 million in 2019 [1, 4]. OA places a huge burden on healthcare services, accounting for 1–2.5% of the gross national product in developed countries, and the cost of these services is expected to quadruple by 2030 [5]. Coupled with the lack of curative clinical treatments [6], the huge growing burden of OA on individuals, families, and healthcare systems drives an urgent need for research on OA and implementation of preventative measures.

Hip OA (HOA) is the second most common form of OA, after knee OA [7]. As with other forms of OA, HOA is characterized by the destruction of articular cartilage and reactive bone changes and clinically correlates with groin pain, joint stiffness, and loss of function [7]. Radiographically, signs of HOA include decreased joint space, marginal osteophyte formation, subchondral cysts, and subchondral sclerosis [8, 9]. For HOA diagnosis, X-ray analysis allows joint space width (JSW) and osteophytes to be assessed non-invasively [9]. Compared to the clinical classification of HOA based on the presence of pain, either self-reported or upon physical examination of the joint, radiographic HOA can better reduce the subjectivity of HOA diagnosis and improve the sensitivity of detection [9]. However, the standards for HOA examinations have remained inconsistent over the years [8, 10–14]. The last systematic review on the prevalence of radiographic HOA, by Dagenais and colleagues in 2009 estimated the global prevalence at 0.9 to 27%, but did not illustrate correlations between risk factors and HOA prevalence [15]. Since then, an estimate of HOA prevalence has not been synthesized at the global level, and subsequent studies still lacked specific and comprehensive analysis on subgroup prevalence estimates, for instance by region, gender, and diagnosis method [7, 10, 15].

An up-to-date understanding of the worldwide prevalence of HOA is imperative for developing effective strategies for primary prevention and management, as well as for informing clinicians and stakeholders on possibly rising healthcare demands. To fill the current gap in understanding the global prevalence of HOA, a systematic review and meta-analysis of epidemiological studies reporting the prevalence of radiographic HOA in the general population were performed. In addition to providing an up-to-date global prevalence of HOA, the study aimed to demonstrate prevalence estimates by subgroup analyses of region, gender, and diagnostic method. Moreover, regression analysis was used to confirm the respective relationships between age and sex, and sex and prevalence.

The protocol for this review was registered on the PROSPERO international prospective register of systematic reviews (http://www.crd.york.ac.uk/PROSPERO) on 7 July 2022 (registration number CRD42022330332).

## Methods

### Search strategy

Published literature reporting the prevalence of radiographic HOA was identified by searching the following databases from inception to 27 August 2022: EMBASE, PubMed, Web of Science, CINAHL, and SCOPUS. “Epidemiology,” “Prevalence,” and “Hip Osteoarthritis” were used as search terms. The full search strategies for different databases are presented in Supplementary Text A1.

The inclusion criteria were as follows: (1) the study was published in English; (2) the study design was cross-sectional or cohort study; (3) the study utilized X-ray to determine the presence of HOA. The exclusion criteria were as follows: (1) the study did not directly report prevalence and did not provide case numbers and sample size that could be used to calculate prevalence; (2) the study included only groups with a particular occupation who had higher likelihood of being predisposed to developing hip-related musculoskeletal disorders (e.g., athletes, farmers, dancers, soldiers); (3) the study only included participants with already diagnosed HOA (hence 100% prevalence); (4) the study had a quality score of less than 4; (5) the study reported data from the same cohort as another study; for multiple published studies using the same cohort, only the study with the highest quality or most complete reporting of information was retained for analysis.

References were imported into Endnote (version X9) and duplicates removed. Two researchers (ZJF and YL) independently screened the titles and abstracts of all retrieved records from the literature search. Full-text articles of studies meeting the selection criteria were retrieved. Consensus was reached for any disagreements through discussion.

### Risk of bias

Included studies were assessed by the Newcastle–Ottawa Scale (NOS) [16] or the Agency for Healthcare Research and Quality (AHRQ) [17]. The NOS scale was used to assess the quality of cohort studies by population selection, comparability, and outcome measures: a score of 0–4 indicated low quality, 5–7 moderate quality, and 8 or more excellent quality. The AHRQ scale was used to assess cross-sectional studies based on 11 items on the list: “Yes” received a score of 1, and “No” and unclear received a score of 0, while the fifth item received a score of 0 for yes or unclear, and 1 for no. A total score of 0 to 3 was considered low quality, 4 to 7 moderate quality, and > 7 high quality [17]. Two authors (ZJF and KNF) independently evaluated the quality of studies and sought advice from a third researcher (JYS) when discrepancies existed.

### Data extraction

All data extraction was performed independently by the same two authors (ZJF and JYS) and any discrepancy resolved by discussion. The following data were extracted from included studies: lead author, study design, quality assessment, continent, patient age, prevalence of HOA, diagnosis method, and cohort.

### Overall pooled prevalence of HOA

HOA prevalence for each study was extracted directly from the article. If the prevalence was not provided, the number of cases divided by the sample size was used (i.e., Prevalence = Cases/Sample size). For studies that did not report total sample size or prevalence, overall prevalence was estimated using reported sex-specific sample sizes and prevalence.

$${Prevalence}=\frac{({p}_{\mathrm{m}}*{N}_{\mathrm{m}}+{p}_{\mathrm{f}}*{N}_{\mathrm{f}})}{{N}_{\mathrm{m}}+{N}_{\mathrm{f}}}$$

(${p}_{\mathrm{m}}$: male prevalence; ${p}_{\mathrm{f}}$: female prevalence; ${N}_{\mathrm{m}}: \mathrm{male sample size};{N}_{\mathrm{f}}: \mathrm{female sample size})$

Before pooling prevalence estimates, the variance of the raw prevalence from each included study was stabilized by using the Freeman-Tukey double arc-sine transformation [18] or logit-transformed proportions [19]. The random effect model was used, and sensitivity analysis was performed to find the source of heterogeneity.

### Subgroup meta-analyses

Subgroup meta-analyses were conducted to examine the differences in HOA prevalence diagnosed using various discriminators. Based on the most frequently used K-L grade classification, the prevalence of radiographic HOA by gender and continents was also integrated.

### Regression analysis

A regression analysis was conducted to determine whether age and gender composition had an effect on prevalence.

### Publication bias

Publication bias in meta-analysis was detected qualitatively by visual inspection of funnel plots and quantitatively by the Egger linear regression test and the Begg rank correlation test when more than 10 estimates were available in a single analysis [20, 21]. *P* < 0.05 (two-tailed) was used as the level of significance. The data were shown as mean with a 95% confidence interval. R (version4.1.2) was used to calculate the prevalence of HOA data.

## Results

### Study selection and assessment

**Fig. 1:**
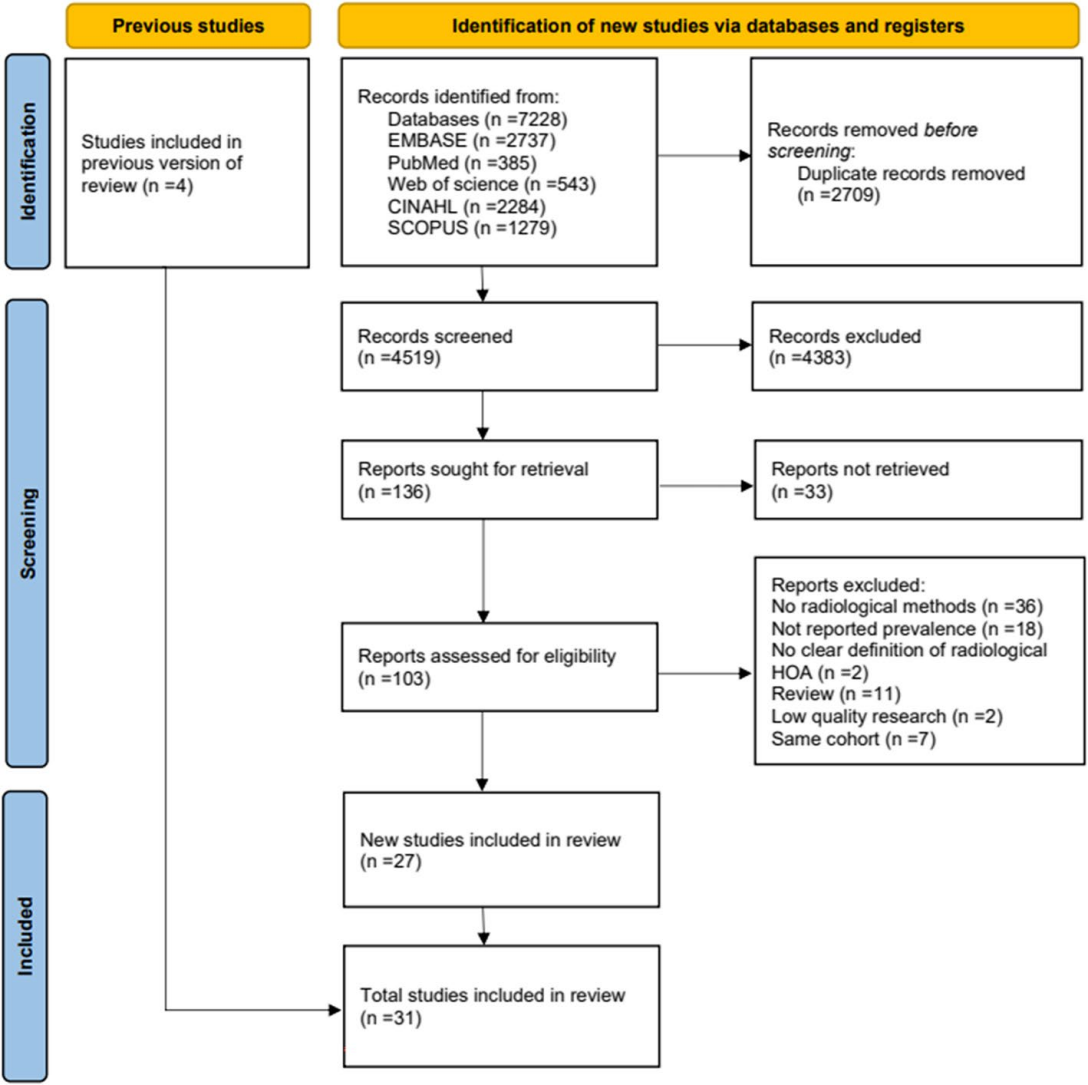
PRISMA diagram showing the study selection process

The initial database search yielded 7228 records. After the removal of duplicates, the title and abstract of 4519 articles were screened and ineligible studies were removed, resulting in 103 articles for full-text screening. Four additional articles were identified through reference searching. The study selection process is presented in Fig. 1. Data on prevalence were extracted from 31 studies, involving 359,251 participants. Quality evaluation showed that all studies included in the analysis had a Quality Score of at least 4 (Table 1) [22–52]. The detailed procedure for study quality evaluation is presented in Supplementary Table A1.

**Table 1:** Characteristics of included studies

Author	Study design	Quality assessment	Continent	Age	Prevalence		Diagnosis method	Cohort
					Participants	Female		
					No. (%)	No. (%)		
Ahlberg, A., 1990 [22]	Cross-sectional	6	Asia	Men 61.4	4721 (0.1)	NR	ACR	NR
Tepper, S., 1993 [23]	Cross-sectional	7	North America	55–74	2358 (3.1)	1202 (3.0)	KL grade ≥ 2	First National Health and Nutrition Examination Survey
Oliveria, S. A., 1995 [24]	Cross-sectional	7	North America	20–89	197,565 (0.1)	100,984 (0.1)	KL grade ≥ 2	the Fallon Community Health Plan
Hirsch, R., 1998 [25]	Cross-sectional	8	North America	45–93	749 (3.6)	457 (2.84)	KL grade ≥ 2	Gila River Indian Community arthritis and diabetes research project
Odding, E., 1998 [26]	Cross-sectional	8	Europe	55–93.2	2895 (15.2)	1739 (15.9)	KL grade ≥ 2	Rotterdam Study
Yoshimura, N., 1998 [27]	Cross-sectional	5	Europe	60–75	1498 (10.2)	195 (4.8)	Croft grade ≥ 3	NR
			Asia	40–79	296 (1.0)	98 (2.0)	Croft grade ≥ 3	
Goker, B., 2001 [28]	Cross-sectional	5	Europe	25–97	566 (11.7)	170 (9.7)	KL grade ≥ 2	NR
Nevitt, M. C., 2002 [29]	Cross-sectional	9	Asia	60–89	1492 (1.0)	878 (1.2)	Other	Beijing OA study
			North America	60–89	7998 (5.5)	7998 (5.5)		Study of Osteoporotic Fractures Study
			North America	60–79	314 (4.2)	158 (3.8)		First National Health and Nutrition Examination Survey
Jacobsen, S., 2004 [30]	Cross-sectional	8	Europe	22–93	3792 (5.8)	2293 (5.9)	JSW ≤ 2	Osteoarthritis Substudy cohort of the third Copenhagen City Heart Study
					3792 (4.7)	2293 (3.7)	KL grade ≥ 2	
					3792 (4.5)	2293 (3.3)	Croft grade ≥ 3	
Lane, N. E., 2004 [31]	Cross-sectional	10	North America	≥ 65	5928 (12.6)	5928 (12.6)	Other	Osteoporotic Fractures Study
Andrianakos, A. A., 2006 [32]	Cross-sectional	9	Europe	46.95 ± 17.74	8740 (0.9)	NR	ACR	The ESORDIG Study
Quintana, J. M., 2008 [33]	Cross-sectional	10	Europe	60–89	1464 (26.5)	934 (26.9)	KL grade ≥ 2	NR
Arden, N. K., 2009 [34]	Cross-sectional	7	North America	70.7 ± 4.7	NR	5839 (2.5)	KL grade ≥ 2	Osteoporotic Fractures Study
						5839 (5.3)	Croft grade ≥ 2	
						5839 (1.8)	Croft grade ≥ 3	
						5839 (3.0)	MJS ≤ 1.5 mm	
						5839 (9.4)	MJS ≤ 2 mm	
						5839 (28.2)	MJS ≤ 2.5 mm	
Chung, C. Y., 2010 [35]	Cross-sectional	9	Asia	65–99	674 (13.1)	386 (15.8)	JSW ≤ 2.5	Korean Longitudinal Study on Health and Aging
					674 (2.1)	386 (2.3)	JSW ≤ 2	
Nelson, A. E., 2010 [36]	Cross-sectional	9	North America	≥ 45	2739 (20.5)	1555 (22.3)	KL grade ≥ 2	Johnston County Osteoarthritis Project
Guillemin, F., 2011 [37]	Cross-sectional	10	Europe	40–75	3707 (8.6)	NR	KL grade ≥ 2	NR
Horváth, G., 2011 [38]	Cross-sectional	7	Europe	20–67	661 (16.5)	NR	KL grade ≥ 2	In the south-western part of Hungary
YefiL, H., 2013 [39]	Cross-sectional	6	Europe	53.9 ± 8.5	522 (1.0)	390 (0.8)	ACR	NR
Kim, C., 2014 [40]	Cross-sectional	7	North America	51–92	978 (18.5)	544 (13.6)	KL grade ≥ 2	Framingham in 2002 – 2005
Barbour, K. E., 2015 [41]	Cohort study	9	North America	71.4 ± 5.1	NR	7889 (8.1)	Croft grade ≥ 2	Osteoporotic Fractures Study
Cho, H. J., 2015 [42]	Cross-sectional	7	Asia	72 ± 5	696 (2.2)	398 (1.8)	KL grade ≥ 2	Korean Longitudinal Study on Health and Aging
Loyola-Sanchez, A., 2016 [43]	Cross-sectional	8	North America	45 ± 18	1479 (0.3)	NR	ACR	NR
Pereira, D., 2016 [44]	Cross-sectional	9	Europe	58.4 ± 14	676 (24.1)	NR	KL grade ≥ 2	EPIPorto cohort
Zhang, J. F., 2016 [45]	Cross-sectional	6	Asia	43.9 ± 16.6	7126 (0.6)	3517 (0.5)	KL grade ≥ 2	NR
Park, J. H., 2017 [46]	Cross-sectional	9	Asia	> 50	8976 (0.6)	5146 (0.3)	KL grade ≥ 2	Korea National Health and Nutrition Examination Surveys
Slimani, S., 2017 [47]	Cross-sectional	4	Africa	15–88	500 (1.2)	NR	KL grade ≥ 2	NR
Vega-Hinojosa, O., 2018 [48]	Cross-sectional	7	South America	18–98	1095 (0.4)	NR	ACR	NR
Damen, J., 2019 [49]	Cross-sectional	7	Europe	55.9 ± 5.2	1002 (32.1)	NR	ACR	Cohort Hip and Cohort Knee
Lidaka, T., 2020 [50]	Cohort study	8	Asia	23–94	2946 (14.9)	1906 (13.2)	KL grade ≥ 2	Osteoarthritis/osteoporosis Against Disability
Macías-Hernández, S. I., 2020 [51]	Cross-sectional	6	North America	57.4 ± 10.9	204 (26.5)	124 (31.5)	KL grade ≥ 2	NR
Costa, Daniela, 2021 [52]	Cross-sectional	8	Europe	> 18	6862 (2.9)	NR	KL grade ≥ 2	EpireumaPt

*K-L* Kellgren and Lawrence, *NR* Not reported, *JSW* Minimum joint space width, *MJS* Minimum hip joint space, *ACR* American College of Rheumatology

### Characteristics of included studies

Two were cohort studies [41, 50] and the others were cross-sectional studies [22–35, 37–40, 42–45, 47–49, 51, 52]. In 4 studies, only women were involved [29, 31, 34, 41]. Out of the 31 studies, 19 (61.3%) used K-L grade ≥ 2 classification to diagnose radiographic HOA [23–26, 28, 30, 33, 34, 36–38, 40, 42, 44, 46, 47, 50–52], while 7 (22.6%) used American College of Rheumatology (ACR) criteria [22, 32, 39, 43, 45, 48, 49], 3 used two or more diagnostic methods [30, 34, 35], and 2 used custom diagnostic methods [29, 31]. A total of 11 studies were from North America (35.5%) [23–25, 29, 31, 34, 36, 40, 41, 43, 51], 7 were from Asia (22.6%) [22, 27, 29, 35, 42, 46, 50], 12 were from Europe (38.7%) [26–28, 30, 32, 33, 37–39, 44, 49, 52], and 1 was from Africa (2.63%) [47]. Comparisons between multiple continents were made in 2 studies [27, 29].

### Pooled and stratified prevalence of HOA

Among the included studies, 7 different radiographic HOA diagnostic methods were used, the most common of which was K-L grade ≥ 2 (*n* = 19). The prevalence of HOA diagnosed by K-L grade ≥ 2 was 5.85% (*k* = 19; 95% CI 2.99–11.15; *I*^2^ = 99.7%), while that by ACR was 0.77% (*k* = 7; 95% CI 0.18–3.16; *I*^2^ = 99.6%), and by Croft grade ≥ 3 was 3.33% (*k* = 3; 95% CI 1.42–7.66;* I*^2^ = 98.5%). There were significant differences in prevalence estimates made using different diagnostic methods (*p* < 0.001) (Table 2). Detailed forest plots are shown in Supplementary Figure A1.

**Table 2:** Prevalence of radiographic HOA using subgroup meta-analysis

Variable	No. of studies	No. of participants	Prevalence, % (95% CI)	*I*^2^, %	*P*-value			
					*Q* test	Subgroup difference	Egger test	Begg test
Diagnosis method subgroup								
Overall	31	326,463	4.01 (2.47–6.43)	99.6	< 0.05	< 0.001	< 0.05	> 0.05
ACR	7	24,685	0.77 (0.18–3.16)	99.6	< 0.05		NR	NR
Croft grade ≥ 2	1	7889	8.05 (7.47–8.67)	-	-		NR	NR
Croft grade ≥ 3	3	11,327	3.33 (1.42–7.66)	98.5	< 0.05		NR	NR
MJS ≤ 1.5 mm	1	5839	3.00 (2.59–3.47)	-	-		NR	NR
MJS ≤ 2 mm	1	5839	9.40 (8.68–10.18)	-	-		NR	NR
MJS ≤ 2.5 mm	1	5839	28.21 (27.07–29.38)	-	-		NR	NR
JSW ≤ 2	2	4466	3.64 (1.76–7.39)	93.1	< 0.05		NR	NR
JSW ≤ 2.5	1	674	13.06 (7.47–15.82)	-	-		NR	NR
K-L grade ≥ 2	19	244,173	5.85 (2.99–11.15)	99.7	< 0.05		< 0.05	> 0.05
Custom^b^	2	15,732	4.23 (1.69–10.19)	99.0	< 0.05		NR	NR
Continent subgroup^a^								
Overall	19	244,173	8.55 (4.85–13.18)	99.5	< 0.05	< 0.001	< 0.05	> 0.05
Asia	3	12,618	4.26 (0.02–14.93)	99.8	< 0.05		NR	NR
North America	7	210,432	7.95 (1.98–17.36)	99.8	< 0.05		NR	NR
Africa	1	500	1.20 (0.44–2.59)	-	-		NR	NR
Europe	8	20,623	12.59 (7.17–19.25)	99.4	< 0.05		NR	NR
Sex subgroup^a^							NR	NR
Overall	14	231,767	8.63 (5.34–12.61)	99.7	< 0.05	> 0.05	< 0. 05	> 0.05
Male	13	108,476	9.42 (4.81–15.34)	99.7	< 0.05		< 0.05	> 0.05
Female	14	123,291	7.94 (3.57–13.81)	99.7	< 0.05		< 0.05	> 0.05

^a^Data analysis based on the K-L grade classification. *ACR* American College of Rheumatology, *K-L* Kellgren and Lawrence, *MJS* Minimum hip joint space, *JSW* minimum joint space width

^b^HOA can be diagnosed if any of the following items are met: (1) a minimum joint space of ≤ 1.5 mm; (2) an osteophyte of grade 2 or higher in any location and either (a) superolateral joint space narrowing of grade 2 or higher or (b) superomedial joint space narrowing of grade 3 or higher; (3) any 3 or more radiographic features of OA; (4) modified Croft grade ≥ 2; (5) MJS ≤ 1.5 mm; (6) JSN superolaterally grade ≥ 2; (7) superomedially grade ≥ 3; (8) osteophyte score ≥ 2

Subsequent analyses were conducted using the K-L grade ≥ 2 samples because this classification system is the most widely used in clinical HOA research. For K-L grade ≥ 2 samples, the estimated HOA prevalence was 1.20% (*k* = 1; 95% CI 0.44–2.59) in Africa, 4.26% (*k* = 3; 95% CI 0.02–14.93; *I*^2^ = 99.8%) in Asia, 7.95% (*k* = 7; 95% CI 1.98–17.36; *I*^2^ = 99.8%) in North America, and 12.59% (*k* = 8; 95% CI 7.17–19.25; *I*^2^ = 99.4%) in Europe. There were significant differences in prevalence estimates between different continents based on the K-L grade ≥ 2 classification (*p* < 0.001). The subgroup meta-analysis showed that the prevalence of HOA was 9.42% (*k* = 13; 95% CI 4.81–15.34; *I*^2^ = 99.7%) in men and 7.94% (*k* = 14; 95% CI 3.57–13.81; *I*^2^ = 99.7%) in women. There was no significant difference in prevalence estimates between different genders based on the K-L grade classification (*p* > 0.05) (Table 2).

### Regression analysis

**Fig. 2:**
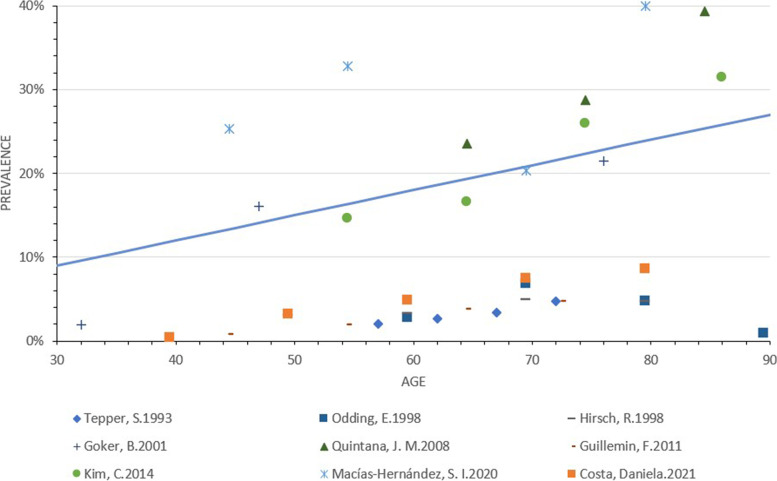
HOA prevalence changes with age among included studies

A total of 9 studies reported HOA prevalence in different age groups [23, 25, 26, 28, 33, 37, 40, 51, 52]. Regression analysis revealed that age was significantly associated with the prevalence of HOA (*p* < 0.05) and explained 11.5% of the variation (Fig. 2). Regression analysis showed no correlation between gender composition and prevalence (*p* > 0.05).

### Sensitivity analysis

Sensitivity analysis was individually performed on each included study to seek sources of heterogeneity, as shown in Supplementary Figure A2. However, the heterogeneity remained high after excluding relevant literature, so the primary results were retained.

### Publication bias

The funnel plot showed that the points were not evenly distributed (Supplementary Figure A3), and the Egger test also showed that there was publication bias (*p* < 0.05). This could be due to different populations and research methods among the included studies.

## Discussion

This systematic review and meta-analysis established the current global prevalence of HOA based on available data. In this analysis, HOA prevalence varied significantly when measured by different diagnostic methods. The most commonly used radiographic diagnosis was K-L grade ≥ 2. On the basis of studies that measured HOA by K-L grade ≥ 2, the prevalence of HOA was highest in Europe and lowest in Africa. Globally, HOA prevalence increased with age. There was no statistical difference in the prevalence of HOA between men and women.

To diagnose HOA, hip radiographs are typically assessed with the K-L grade classification, which focuses on the presence of bone fragments and hip joint space [11, 53]. ACR and MJS criteria are two other frequently used radiographic HOA diagnosis techniques [9]. The ACR criteria emphasize hip pain, which are not appropriate for the early diagnosis of HOA [49]. The MJS criteria may overstate HOA prevalence in women since the minimal joint space in women is less than that in men [54]. The K-L grade was the most commonly used diagnosis method among our included studies. Therefore, studies based on the K-L grade classification were selected for subsequent subgroup meta-analysis on continents and gender.

The findings indicated that HOA was less prevalent in Asia compared to North America and Europe, which was consistent with previous studies [42, 44]. Some possible causes include the following: (1) genetic differences, where people from Western countries tend to have greater build and higher average weight than people from Asian countries [55], since weight has strong correlation with the incidence of OA [10]. (2) Anatomical differences, such as a lower rate of acetabular dysplasia in people from Asian countries [27] and a deeper size of the acetabulum in people from European countries [27], possibly both accounting for a relatively higher prevalence of HOA in Europe. (3) Lifestyle differences, with people from Western countries having greater likelihood of consuming a high-fat or high-sugar diet, as well as greater tendency to engage in high-risk or high-impact sports compared to people from Asian countries [55]. In line with previous systematic reviews and individual investigations, a positive association between age and the prevalence of HOA was found [23, 28, 33, 37, 40, 52]. This finding confirms that increasing age may be a risk factor for HOA.

No significant difference in HOA prevalence was found between men and women, which is consistent with previous literature reporting findings on an Indian population and from urography measurements [25, 28], respectively. Nevertheless, other studies have reported that women are at higher risk of developing HOA [12, 42, 56, 57]. The distribution of age may be relevant to the risk of HOA. This research spanned a wide age range, which may have offset the gender-based differences in HOA prevalence in the aged population since HOA becomes more common in women than men after menopause. Several studies support this hypothesis, which have reported a protective effect of estrogen replacement therapy against HOA [36, 51, 58]. Moreover, men are more likely than women to develop osteochondrosis and subchondral sclerosis, which increases the prevalence of HOA in men by the K-L grade classification emphasizing osteochondrosis and joint space [30]. More studies are needed to more precisely characterize the association between gender and HOA.

There is still a lack of consensus in defining HOA. The establishment of a gold standard definition based on radiographic diagnosis requires consideration of the following questions: (1) Does it apply to different stages of HOA? It is important to consider that the pathologic features and symptoms of OA can occur before the disease is detected on radiographs [8]. (2) How are the imaging sites selected? Some studies have shown that colon, pelvic, or abdominal radiographs can easily detect HOA in pain-free patients, but can affect the accuracy of results and inflate prevalence [15, 35]. (3) Is there a gold standard that applies to both men and women? The diagnostic method needs to consider gender-based differences, for instance, men are more likely than women to develop osteochondrosis and subchondral sclerosis [30].

Compared with previous studies relating to radiographic prevalence of HOA [7, 15, 57], the study has the following strengths and differences: (1) The study performed a more thorough search using a total of five databases and included 8 newer studies in its analysis, compared to the previous most recent systematic review on this topic which used two databases [15]. (2) The study was the first to identify differences in HOA prevalence globally that were separated into geographic regions and diagnostic methods, which is of great significance for the regionalized diagnosis and prevention of HOA. (3) The study established the relationship between age and HOA prevalence. This finding highlights the need to prioritize the prevention and treatment of HOA for the elderly. (4) The study found no statistical difference in HOA prevalence between men and women, which is consistent with previous studies despite other literature findings indicating an increased risk in HOA in elderly women. The finding in this study therefore has great significance in directing further investigation on this topic.

### Limitations

The interpretation of findings presented in this review is subject to a few limitations: (1) This study was limited to X-ray diagnosis of HOA, which might have resulted in fewer studies being included and thus lower confidence. (2) The high heterogeneity of the study findings and the existence of publication bias require caution in the interpretation of results. (3) This research only included studies published in the English language, which may have introduced some selection bias, although this limitation is offset by the inclusion of OA cohorts from different countries and regions. (4) The prevalence in Africa is based on a single study, which may not be representative of the overall population.

Based on the findings, this study advocates for health professionals, decision-makers, and the general public to gain better awareness of HOA and its impacts on global healthcare systems. There is an urgent need to develop a gold standard for the identification of radiographic HOA. Sizeable differences in the prevalence of radiographic HOA across regions call for an in-depth investigation to unpack the drivers and risk factors of HOA at systemic levels, as well as to develop more effective interventions and management strategies.

## Conclusion

Globally, the radiographic prevalence of HOA is the highest in Europe at 12.59% and the lowest in Africa at 1.2%. There is no statistically significant difference in HOA prevalence between men and women. Age is a positively significant influencing factor of HOA prevalence. The findings point to the need for more high-quality epidemiological studies in the future on the prevalence of HOA, particularly in underrepresented regions such as Africa, Oceania, and South America.

## Supplementary Information

**Additional file 1.**

## Data Availability

The guarantor (BW) is willing to examine all requests for the full dataset after a period of 2 years from the date of this publication. The corresponding author should be contacted at BW. wangbin_pku@zju.edu.cn. The lead author (BW) affirms that the manuscript is an honest, accurate, and transparent account of the study being reported; that no important aspects of the study have been omitted; and that any discrepancies from the study as planned (and, if relevant, registered) have been explained.

## Abbreviations

OA: Osteoarthritis
HOA: Hip osteoarthritis
JSW: Joint space width
NOS: Newcastle–Ottawa Scale
AHRQ: Agency for Healthcare Research and Quality
K-L: Kellgren and Lawrence
NR: Not reported
MJS: Minimum hip joint space
ACR: American College of Rheumatology
