# Supplementary material for: The prevalence of hip osteoarthritis: a systematic review and meta-analysis
Source: Arthritis Res Ther. 2023 Mar 29;25:51. doi: 10.1186/s13075-023-03033-7 (PMC10053484; doi:10.1186/s13075-023-03033-7)
Supplement: Supplementary file 1 — Additional file 1. [file 13075_2023_3033_MOESM1_ESM.docx]

| **The prevalence of radiographic hip osteoarthritis: A systematic review and Meta‑Analysis** |
| --- |

**Contents**

[Text A1: Search strategies 2](#_Toc113304577)

[Table A1: Quality evaluation results 7](#_Toc113304578)

[Table A1.1: AHRQ quality evaluation results 7](#_Toc113304579)

[Table A1.2: NOS quality evaluation results 11](#_Toc113304580)

[Figure A1: Forest plot 12](#_Toc113304581)

[Figure A1.1: Hip Osteoarthritis prevalence for Diagnosis method 12](#_Toc113304582)

[Figure A1.2: Hip Osteoarthritis prevalence for region 13](#_Toc113304583)

[Figure A1.3: Hip Osteoarthritis prevalence for gender 14](#_Toc113304584)

[Figure A2: Sensitivity analysis results 15](#_Toc113304585)

[Figure A2.1: Hip Osteoarthritis prevalence by Diagnosis method 15](#_Toc113304586)

[Figure A2.2: Hip Osteoarthritis prevalence by region 16](#_Toc113304587)

[Figure A2.3: Hip Osteoarthritis prevalence by gender 17](#_Toc113304588)

[Figure A3: Funnel plot 18](#_Toc113304589)

[Figure A3.1: Hip Osteoarthritis prevalence by Diagnosis method 18](#_Toc113304590)

[Figure A3.2: Hip Osteoarthritis prevalence by K&L grade 19](#_Toc113304591)

[Figure A3.3: Hip Osteoarthritis prevalence by region 20](#_Toc113304592)

[Figure A3.4: Hip Osteoarthritis prevalence by gender 21](#_Toc113304593)

[Figure A3.5: Hip Osteoarthritis prevalence by male 22](#_Toc113304594)

[Figure A3.6: Hip Osteoarthritis prevalence by female 23](#_Toc113304595)

## Text A1: Search strategies

PubMed

|  | Search term | | Results |
| --- | --- | --- | --- |
| #25 | #23 and #24 |  | 385 |
| #24 | #6 OR #15 |  | 488118 |
| #23 | #16 OR #17 OR #18 OR #19 OR #20 OR #21 OR #22 | | 12391 |
| #22 | Osteoarthritis of the Hip[Title/Abstract] | | 1716 |
| #21 | Coxarthroses[Title/Abstract] | | 89 |
| #20 | Coxarthrosis[Title/Abstract] | | 1599 |
| #19 | Osteoarthritis Of Hips[Title/Abstract] | | 7 |
| #18 | Osteoarthritis Of Hip[Title/Abstract] | | 775 |
| #17 | Hip Osteoarthritis[Title/Abstract] | | 2991 |
| #16 | Osteoarthritis, Hip[MeSH Terms] | | 9503 |
| #15 | #7 OR #8 OR #9 OR #10 OR #11 OR #12 OR #13 OR #14 | | 346012 |
| #14 | Prevalence, Point[Title/Abstract] | | 33 |
| #13 | Point Prevalences[Title/Abstract] | | 113 |
| #12 | Point Prevalence [Title/Abstract] | | 5906 |
| #11 | Prevalence, Period[Title/Abstract] | | 56 |
| #10 | Period Prevalences[Title/Abstract] | | 64 |
| #9 | Period Prevalence[Title/Abstract] | | 1579 |
| #8 | Prevalences[Title/Abstract] | | 15802 |
| #7 | Prevalence[MeSH Terms] | | 333651 |
| #6 | #1 OR #2 OR #3 OR #4 OR #5 | | 160309 |
| #5 | Social Epidemiologies[Title/Abstract] | | 1 |
| #4 | Epidemiology, Social[Title/Abstract] | | 65 |
| #3 | Epidemiologies, Social[Title/Abstract] | | 132886 |
| #2 | Social Epidemiology[Title/Abstract] | | 832 |
| #1 | Epidemiology[MeSH Terms] | | 28166 |

Embase

|  | Search term | Results |
| --- | --- | --- |
| #11 | #9 AND #10 | 2737 |
| #10 | #7 OR #8 | 4181172 |
| #9 | #3 OR #6 | 16051 |
| #8 | #2 OR #5 | 914910 |
| #7 | #1 OR #4 | 4174817 |
| #6 | 'osteoarthritis, hip':ti,ab,kw OR 'osteoarthritis of hip':ti,ab,kw OR 'osteoarthritis of hips':ti,ab,kw OR coxarthrosis:ti,ab,kw OR coxarthroses:ti,ab,kw OR 'osteoarthritis of the hip':ti,ab,kw | 4619 |
| #5 | 'prevalences':ti,ab,kw OR 'period prevalence':ti,ab,kw OR 'period prevalences':ti,ab,kw OR 'prevalence, period':ti,ab,kw OR 'point prevalence':ti,ab,kw OR 'point prevalences':ti,ab,kw OR 'prevalence, point':ti,ab,kw | 29172 |
| #4 | 'social epidemiology':ti,ab,kw OR 'epidemiologies, social':ti,ab,kw OR 'epidemiology, social':ti,ab,kw OR 'social epidemiologies':ti,ab,kw | 1039 |
| #3 | 'hip osteoarthritis'/exp | 1383841 |
| #2 | 'prevalence'/exp | 905865 |
| #1 | 'epidemiology'/exp | 4174318 |

Web Of Science

|  | Search term | | Results |
| --- | --- | --- | --- |
| #23 | | #21 and #22 | 543 |
| #22 | | #4 OR #13 | 1214818 |
| #21 | | #14 OR #15 OR #16 OR #17 OR #18 OR #19 OR #20 | 4595 |
| #20 | | TI =(Osteoarthritis Of the Hip) | 1882 |
| #19 | | TI =(Coxarthroses) | 9 |
| #18 | | TI =(Coxarthrosis) | 335 |
| #17 | | TI =(Osteoarthritis Of Hips) | 3190 |
| #16 | | TI =(Osteoarthritis Of Hip) | 3190 |
| #15 | | TI=(Hip Osteoarthritis) | 4254 |
| #14 | | TI =(Osteoarthritis ,Hip) | 4254 |
| #13 | | #5 OR #6 OR #7 OR #8 OR #9 OR #10 OR #11 OR #12 | 927044 |
| #12 | | TI=(Prevalence,Point) | 1 |
| #11 | | TI=(Point Prevalences) | 980 |
| #10 | | TI =(Point Prevalence) | 980 |
| #9 | | TI =(Prevalence,Period) | 4 |
| #8 | | TI =(Period Prevalences) | 650 |
| #7 | | TI=(Period Prevalence) | 650 |
| #6 | | TI=(Prevalences) | 180703 |
| #5 | | TS=(Prevalence) | 927044 |
| #4 | | #1 OR #2 OR #3 | 383652 |
| #3 | | TI =(Epidemiology, Social) | 425 |
| #2 | | TI=(Social Epidemiology) | 425 |
| #1 | | TS=(Epidemiology) | 383652 |

CINAHL

|  | Search term | | Results |
| --- | --- | --- | --- |
| #1 | SU Epidemiology | 2126537 | |
| #2 | AB social epidemiology OR TI social epidemiology | 1989 | |
| #3 | AB Epidemiologies, Social OR TI Epidemiologies, Social | 1989 | |
| #4 | S1 OR S2 OR S3 | 2127601 | |
| #5 | SU Prevalence | 415844 | |
| #6 | TI Prevalences OR AB Prevalences | 117986 | |
| #7 | TI period prevalence OR AB period prevalence | 10809 | |
| #8 | TI Prevalence, Period OR AB Prevalence, Period | 10809 | |
| #9 | TI Prevalence, Point OR AB Prevalence, Point | 12739 | |
| #10 | TI Point Prevalences OR AB Point Prevalences | 12739 | |
| #11 | S5 OR S6 OR S7 OR S9 OR S10 | 1278083 | |
| #12 | S4 OR S11 | 2936562 | |
| #13 | SU osteoarthritis hip | 9847 | |
| #14 | TI Hip Osteoarthritis OR AB Hip Osteoarthritis | 11594 | |
| #15 | TI Osteoarthritis Of Hip OR AB Osteoarthritis Of Hip | 10365 | |
| #16 | TI Coxarthrosis OR AB Coxarthrosis | 1928 | |
| #17 | S13 OR S14 OR S15OR S16 | 17231 | |
| #18 | S12 AND S17 | 2284 | |

Scopus

|  | Search term | Results |
| --- | --- | --- |
|  | ( ( ( ( KEY ( prevalence ) ) OR ( TITLE-ABS-KEY ( prevalences ) ) ) OR ( TITLE-ABS-KEY ( "Period Prevalences" ) ) OR ( TITLE-ABS-KEY ( "Point Prevalence" ) ) OR ( TITLE-ABS-KEY ( "Prevalence, Point" ) ) ) OR ( ( KEY ( epidemiology ) ) OR ( TITLE-ABS-KEY ( "social epidemiology " ) ) ) ) AND ( ( ( KEY ( "Hip Osteoarthritis" ) ) OR ( TITLE-ABS-KEY ( "Osteoarthritis, Hip" ) ) ) OR ( ( TITLE-ABS-KEY ( coxarthrosis ) ) OR ( TITLE-ABS-KEY ( coxarthroses ) ) ) OR ( TITLE-ABS-KEY ( "Osteoarthritis of the Hip" ) ) ) | 1279 |

## Table A1: Quality evaluation results

### Table A1.1:Agency for Healthcare Research and Quality(AHRQ)[1] quality evaluation results

| Study | Item 1 | Item 2 | Item 3 | Item 4 | Item 5 | Item 6 | Item 7 | Item 8 | Item 9 | Item 10 | Item 11 | Over | |
| --- | --- | --- | --- | --- | --- | --- | --- | --- | --- | --- | --- | --- | --- |
| Ahlberg, A.1990 | Yes | Yes | Yes | Yes | Unclear | Yes | Yes | No | No | No | Unclear | 6 |  |
| Andrianakos,A. A.2006 | Yes | Yes | Yes | Yes | No | Yes | Yes | Yes | No | Yes | Unclear | 9 |  |
| Costa, Daniela.2021 | Yes | Yes | Yes | Yes | No | Yes | Yes | Yes | No | No | Unclear | 8 |  |
| Damen, J.2019 | Yes | Yes | Yes | Yes | No | Yes | Yes | No | No | No | Unclear | 7 |  |
| Goker, B.2001 | Yes | No | Yes | Yes | Unclear | No | Yes | No | No | No | No | 5 |  |
| Guillemin, F.2011 | Yes | Yes | Yes | Yes | No | Yes | Yes | Yes | Yes | No | Unclear | 10 |  |
| Hirsch, R.1998 | Yes | Yes | Yes | Yes | No | Yes | Yes | Yes | Unclear | No | Unclear | 8 |  |
| Jacobsen, S.2004 | Yes | Yes | Yes | Yes | No | Yes | Yes | Yes | No | No | Unclear | 8 |  |
| Lane, N. E.2004 | Yes | Yes | Yes | Yes | No | Yes | Yes | Yes | Unclear | Yes | Yes | 10 |  |
| Nelson, A. E.2010 | Yes | Yes | Yes | Yes | No | Yes | Yes | Yes | Unclear | Yes | Unclear | 9 |  |
| Nevitt, M. C.2002 | Yes | Yes | Yes | Yes | No | Yes | Yes | Yes | Unclear | Yes | Unclear | 9 |  |
| Odding, E.1998 | Yes | Yes | Yes | Yes | No | Yes | Yes | Yes | Unclear | No | Unclear | 8 |  |
| Oliveria, S. A.1995 | Yes | Yes | Yes | Yes | No | Yes | No | Yes | Unclear | No | Unclear | 7 |  |
| Park, J. H.2017 | Yes | Yes | Yes | Yes | No | Yes | Yes | Yes | Unclear | Yes | Unclear | 9 |  |
| Pereira, D.2016 | Yes | Yes | Yes | Yes | No | Yes | Yes | Yes | Unclear | Yes | Unclear | 9 |  |
| Tepper, S.1993 | Yes | Yes | Yes | Yes | No | Yes | Yes | No | Unclear | No | Unclear | 7 |  |
| YefiL, H.2013 | Yes | Yes | Yes | Yes | No | Yes | No | No | No | No | Unclear | 6 |  |
| Zhang, J. F.2016 | Yes | Yes | Yes | Yes | No | Yes | No | No | Unclear | No | Unclear | 6 |  |
| Arden, Nigel K.2009 | Yes | Yes | Yes | Yes | No | Yes | Yes | No | Unclear | No | Unclear | 7 |  |
| Chung, C. Y.2010 | Yes | Yes | Yes | Yes | No | Yes | Yes | Yes | Unclear | Yes | Unclear | 9 |  |
| Horvath, Gabor2011 | Yes | Yes | Yes | Yes | No | Yes | Yes | No | Unclear | No | Unclear | 7 |  |
| Cho, H. J.2015 | Yes | Yes | Yes | Yes | No | Yes | Yes | No | No | No | Unclear | 7 |  |
| Yoshimura, N.1998 | Yes | Yes | Yes | Yes | No | No | No | No | Unclear | No | Unclear | 5 |  |
| Kim, C.2014 | Yes | Yes | Yes | Yes | No | Yes | Yes | No | Unclear | No | Unclear | 7 |  |
| Quintana, J. M.2008 | Yes | Yes | Yes | Yes | No | Yes | Yes | Yes | Yes | Yes | Unclear | 10 |  |
| Macías-Hernández, S. I.2020 | Yes | Yes | Yes | Yes | No | No | No | Yes | Unclear | No | Unclear | 6 |  |
| Loyola-Sanchez, A.2016 | Yes | Yes | Yes | Yes | No | No | Yes | Yes | No | Yes | Unclear | 8 |  |
| Slimani, S.2017 | Yes | No | Yes | Yes | No | No | No | No | No | No | Unclear | 4 |  |
| Vega-Hinojosa, O.2018 | Yes | Yes | Yes | Yes | No | No | Yes | No | No | Yes | Unclear | 7 |  |

### **Table A1.2:T**he Newcastle–Ottawa Scale (NOS)[2] quality evaluation results

|  | Selection | | Comparability | | Outcome | | All |
| --- | --- | --- | --- | --- | --- | --- | --- |
| Barbour, K. E.2015 | | 4 | | 2 | | 3 | 9 |
| Lidaka, T.2020 | | 4 | | 1 | | 3 | 8 |

[1] Rostom A, Dubé C, and Cranney A, "Celiac Disease. Rockville (MD): Agency for Healthcare Research and Quality (US)," p. Appendix D. Quality Assessment Forms. Available from: <https://www.ncbi.nlm.nih.gov/books/NBK35156/>, 2004.

[2] A. Stang, "Critical evaluation of the Newcastle-Ottawa scale for the assessment of the quality of nonrandomized studies in meta-analyses," (in eng), *European Journal of Epidemiology,* vol. 25, no. 9, pp. 603-605, 2010.

## Figure A1: Forest plot

### Figure A1.1: Hip Osteoarthritis prevalence for Diagnosis method


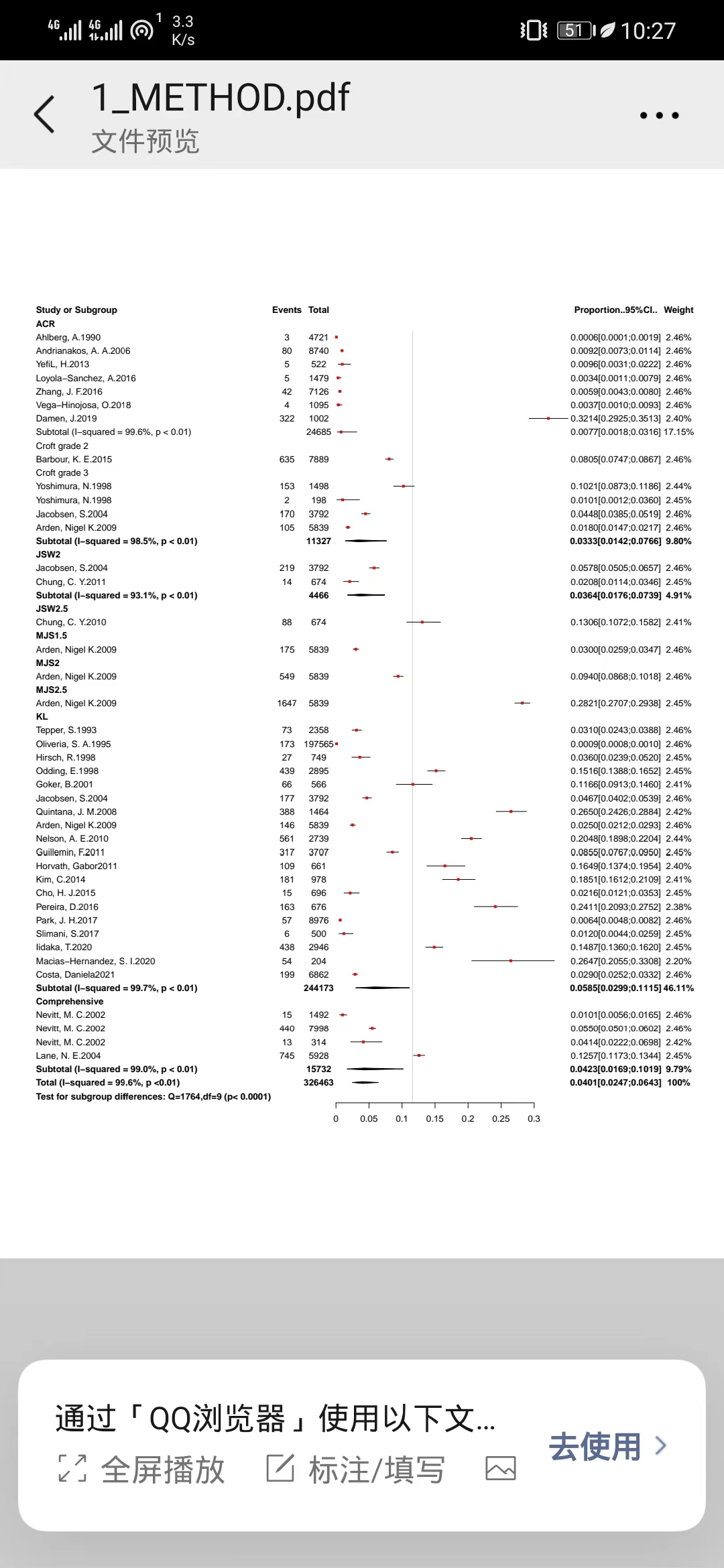


### Figure A1.2: Hip Osteoarthritis prevalence for region


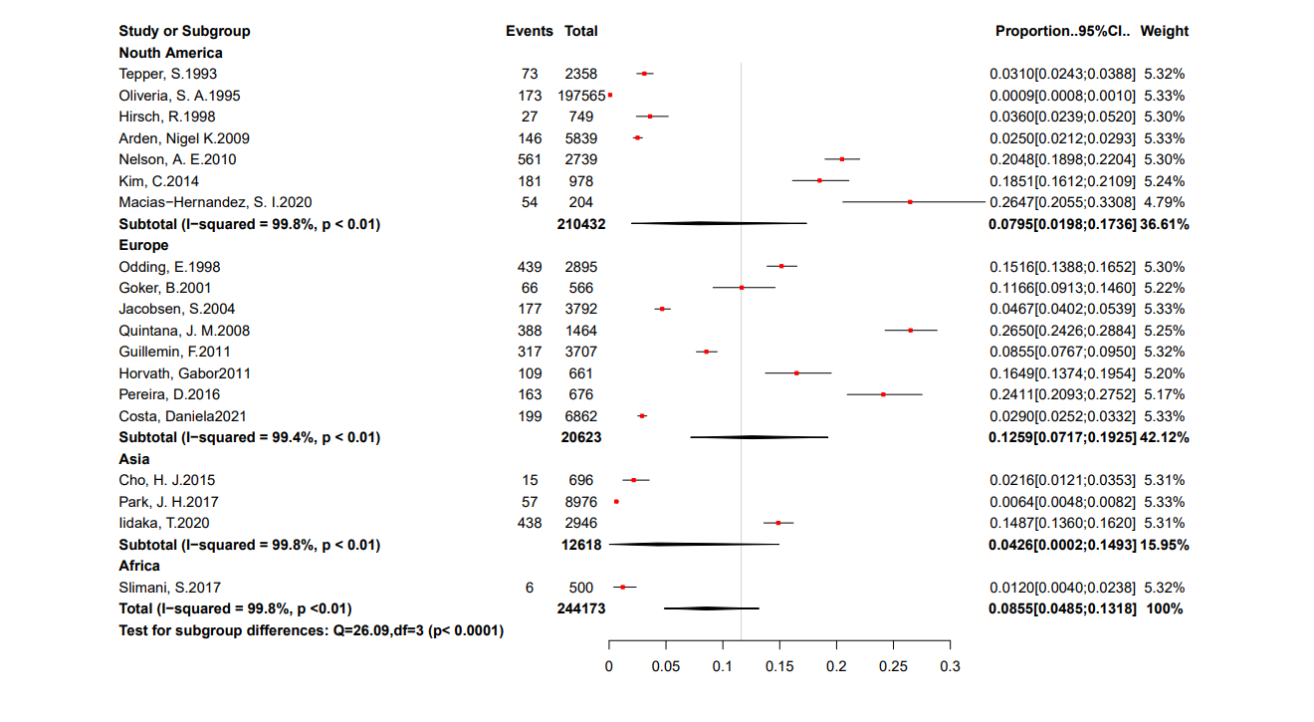


### Figure A1.3: Hip Osteoarthritis prevalence for gender


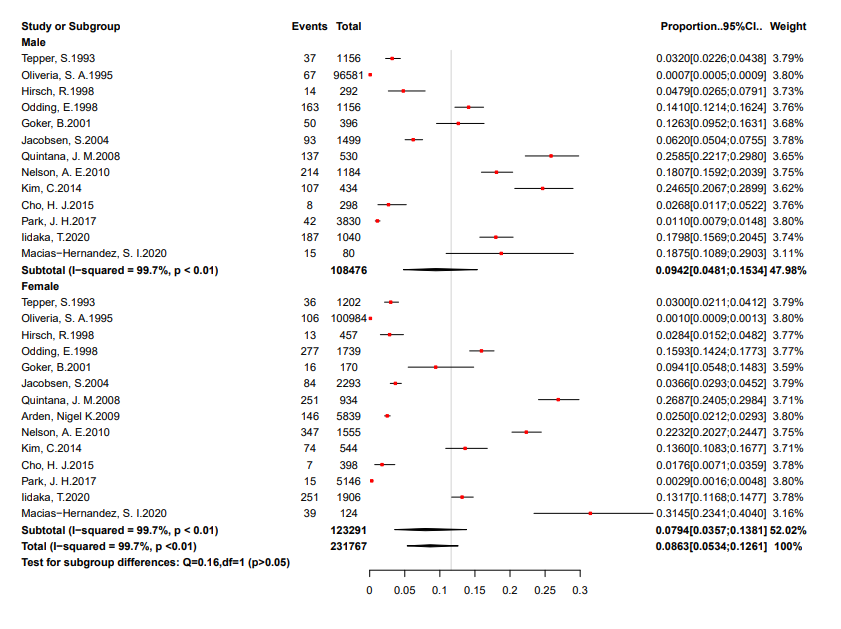


## Figure A2: Sensitivity analysis results

### Figure A2.1: Hip Osteoarthritis prevalence by Diagnosis method


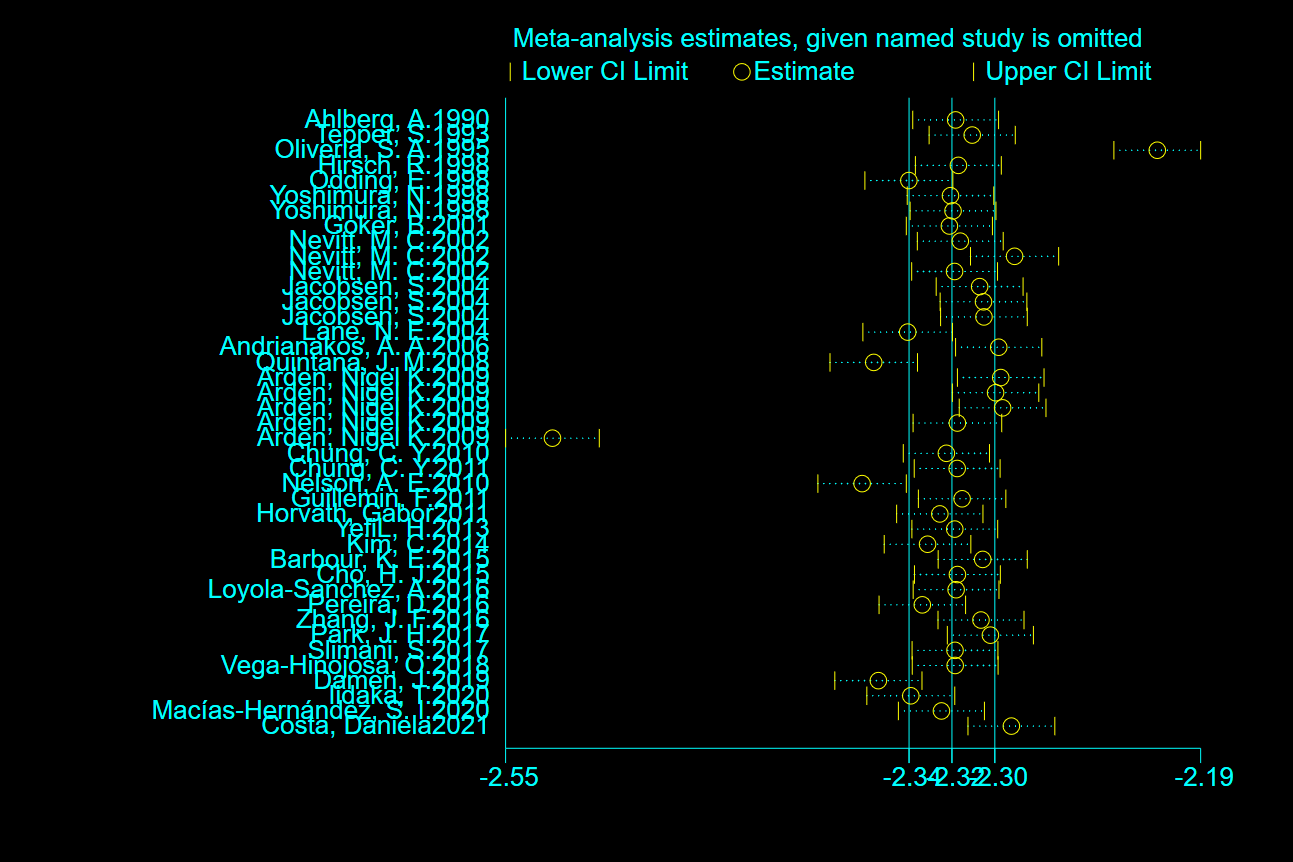


**Logit transformation**

### Figure A2.2: Hip Osteoarthritis prevalence by region


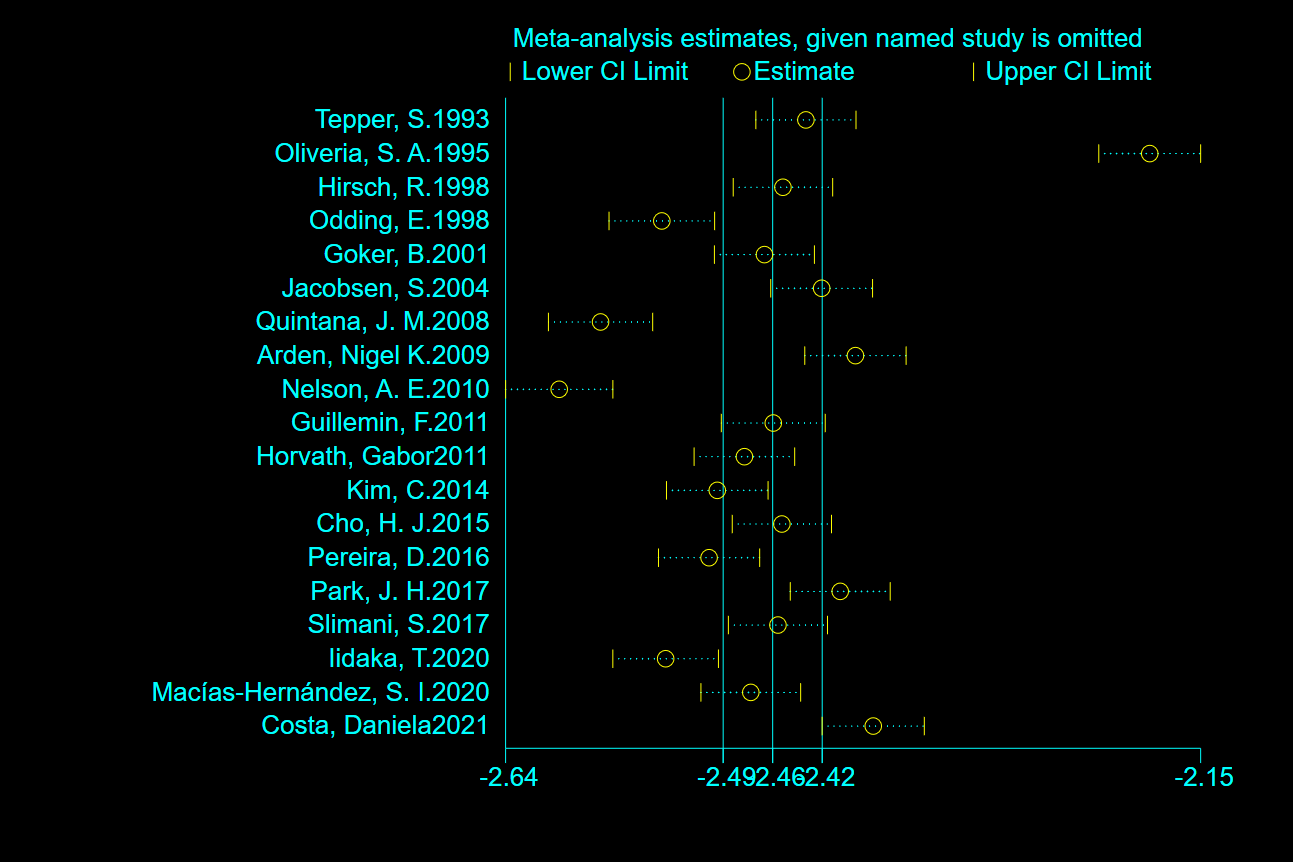


**Freeman-Tukey double arcsine transformation**

### Figure A2.3: Hip Osteoarthritis prevalence by gender


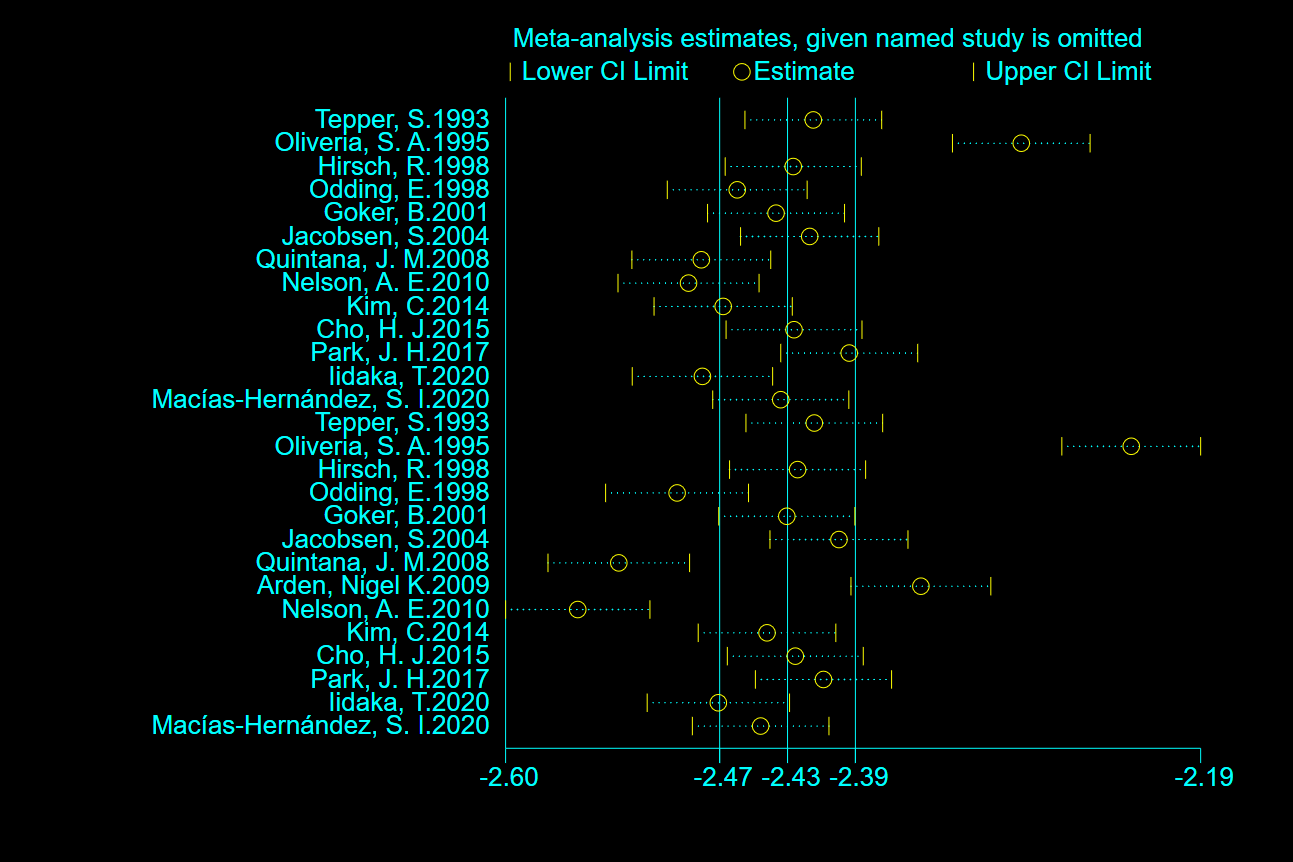


**Freeman-Tukey double arcsine transformation**

## Figure A3: Funnel plot

### Figure A3.1: Hip Osteoarthritis prevalence by Diagnosis method


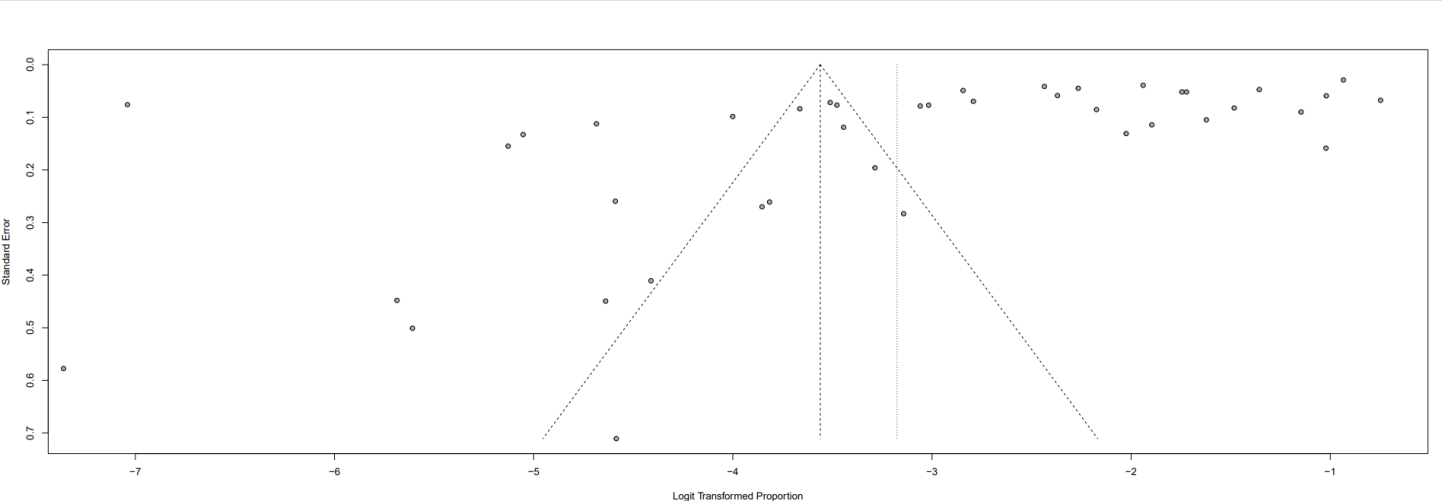


**Logit transformation**

### Figure A3.2: Hip Osteoarthritis prevalence by K&L grade


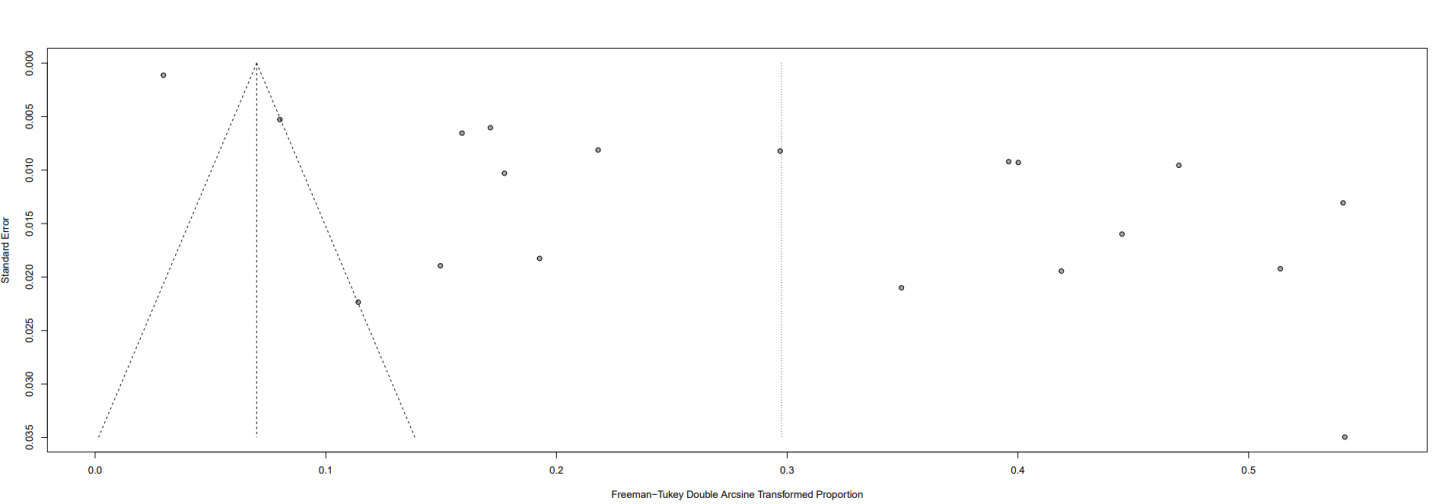


**Freeman-Tukey double arcsine transformation**

### Figure A3.3: Hip Osteoarthritis prevalence by region


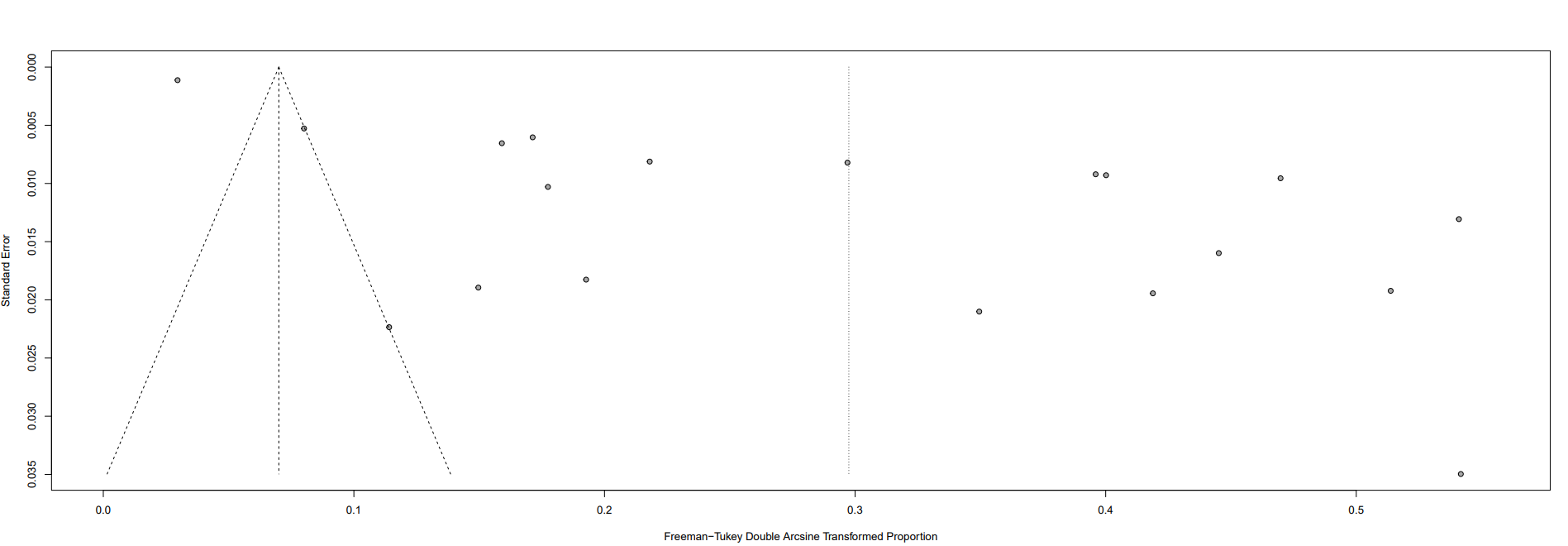


**Freeman-Tukey double arcsine transformation**

### Figure A3.4: Hip Osteoarthritis prevalence by gender


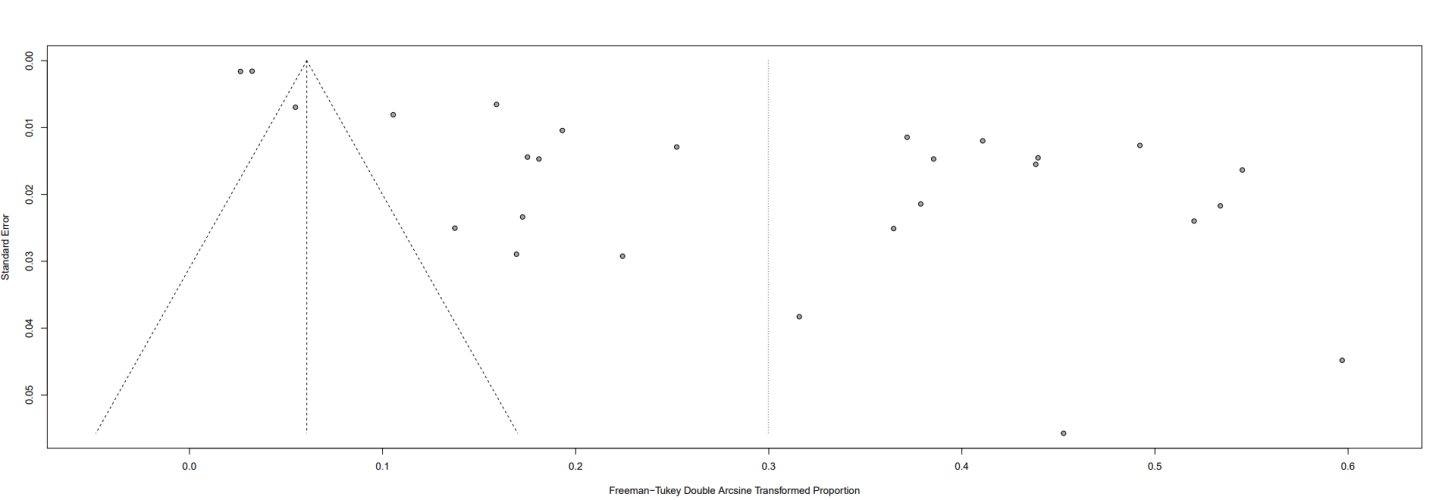


**Freeman-Tukey double arcsine transformation**

### Figure A3.5: Hip Osteoarthritis prevalence by male


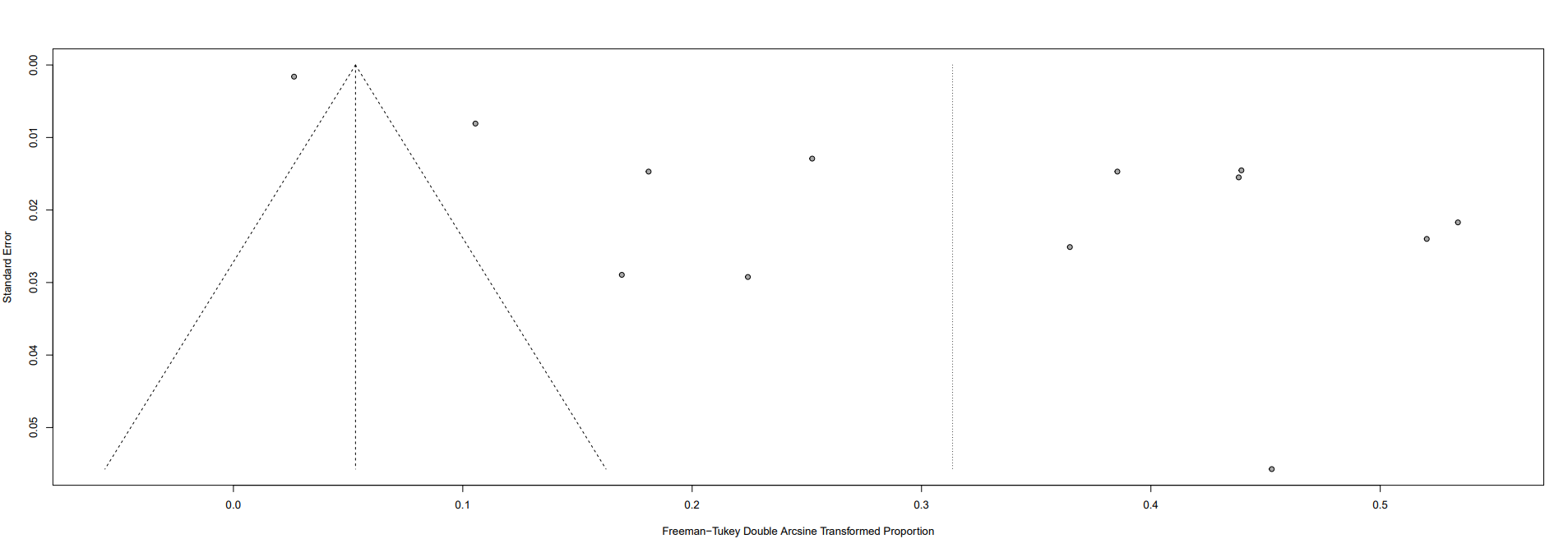


**Freeman-Tukey double arcsine transformation**

### Figure A3.6: Hip Osteoarthritis prevalence by female


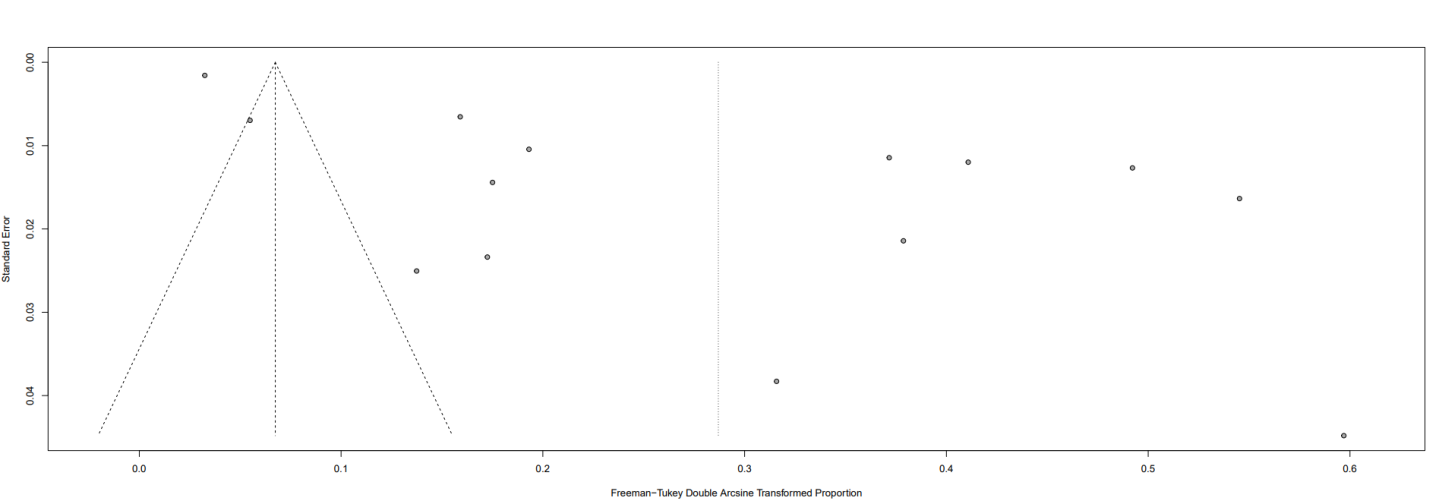


**Freeman-Tukey double arcsine transformation**
